# Shedding of soluble glycoprotein 1 detected during acute Lassa virus infection in human subjects

**DOI:** 10.1186/1743-422X-7-306

**Published:** 2010-11-09

**Authors:** Luis M Branco, Jessica N Grove, Lina M Moses, Augustine Goba, Mohammed Fullah, Mambu Momoh, Randal J Schoepp, Daniel G Bausch, Robert F Garry

**Affiliations:** 1 Tulane University Health Sciences Center, New Orleans, LA, USA; 2 Autoimmune Technologies, LLC, New Orleans, LA, USA; 3 Tulane University School of Public Health & Tropical Medicine, New Orleans, LA, USA; 4 Applied Diagnostics Branch, U.S. Army Medical Research Institute of Infectious Diseases Diagnostic Systems Division, Fort Detrick, Frederick, MD, USA; 5 Lassa Fever Laboratory - Kenema Government Hospital, Kenema, Sierra Leone

## Abstract

**Background:**

Lassa hemorrhagic fever (LHF) is a neglected tropical disease with significant impact on the health care system, society, and economy of Western and Central African nations where it is endemic. With a high rate of infection that may lead to morbidity and mortality, understanding how the virus interacts with the host's immune system is of great importance for generating vaccines and therapeutics. Previous work by our group identified a soluble isoform of the Lassa virus (LASV) GP1 (sGP1) *in vitro *resulting from the expression of the glycoprotein complex (GPC) gene [1,2]. Though no work has directly been done to demonstrate the function of this soluble isoform in arenaviral infections, evidence points to immunomodulatory effects against the host's immune system mediated by a secreted glycoprotein component in filoviruses, another class of hemorrhagic fever-causing viruses. A significant fraction of shed glycoprotein isoforms during viral infection and biogenesis may attenuate the host's inflammatory response, thereby enhancing viral replication and tissue damage. Such shed glycoprotein mediated effects were previously reported for Ebola virus (EBOV), a filovirus that also causes hemorrhagic fever with nearly 90% fatality rates [3-5]. The identification of an analogous phenomenon *in vivo *could establish a new correlate of LHF infection leading to the development of sensitive diagnostics targeting the earliest molecular events of the disease. Additionally, the reversal of potentially untoward immunomodulatory functions mediated by sGP1 could potentiate the development of novel therapeutic intervention. To this end, we investigated the presence of sGP1 in the serum of suspected LASV patients admitted to the Kenema Government Hospital (KGH) Lassa Fever Ward (LFW), in Kenema, Sierra Leone that tested positive for viral antigen or displayed classical signs of Lassa fever.

**Results:**

It is reasonable to expect that a narrow window exists for detection of sGP1 as the sole protein shed during early arenaviral biogenesis. This phenomenon was clearly distinguishable from virion-associated GP1 only prior to the emergence of *de novo *viral particles. Despite this restricted time frame, in 2/46 suspected cases in two studies performed in late 2009 and early 2010, soluble glycoprotein component shedding was identified. Differential detection of viral antigens GP1, GP2, and NP by western blot yielded five different scenarios: whole LASV virions (GP1, GP2, NP; i.e. active viremia), different combinations of these three proteins, sGP1 only, NP only, and absence of all three proteins. Four additional samples showed inconclusive evidence for sGP1 shedding due to lack of detection of GP2 and NP by western blot; however, a sensitive LASV NP antigen capture ELISA generated marginally positive signals

**Conclusions:**

During a narrow window following active infection with LASV, soluble GP1 can be detected in patient sera. This phenomenon parallels other VHF infection profiles, with the actual role of a soluble viral glycoprotein component *in vivo *remaining largely speculative. The expenditure of energy and cellular resources toward secretion of a critical protein during viral biogenesis without apparent specific function requires further investigation. Future studies will be aimed at systematically identifying the role of LASV sGP1 in the infection process and outcome *in vitro *and *in vivo*.

## Background

Lassa virus, a member of the *Arenaviridae *family, is the etiologic agent of Lassa fever, which is an acute and often fatal illness endemic to West Africa. There are an estimated 300,000 - 500,000 cases of Lassa fever each year, with a mortality rate of 15% - 20% for hospitalized patients and as high as 50% during epidemics [9,10]. Presently, there is no licensed vaccine or immunotherapy available for preventing or treating this disease. Although the antiviral drug ribavirin is somewhat beneficial, it must be administered at an early stage of infection to successfully alter disease outcome, thereby limiting its utility [11]. Furthermore, there is no commercially available Lassa fever diagnostic assay, thereby inhibiting early detection and rapid implementation of existing treatment regimens (e.g. ribavirin administration). The lack of adequate countermeasures and means of detection, coupled with the severity of disease, contributed to the classification of LASV as a National Institutes of Allergy and Infectious Diseases (NIAID) Category A pathogen and biosafety level-4 (BSL-4) agent. Presently, the Hemorrhagic Fever Virus Diagnostics Consortium is developing and implementing highly sensitive and specific next generation recombinant diagnostic assays that will be easily deployed and analyzed in remote locations of endemic areas with few laboratory resources.

The LASV genome is comprised of two ambisense, single-stranded RNA molecules designated small (S) and large (L) [12]. Two genes on the S segment encode the nucleoprotein (NP) and a precursor glycoprotein complex (GPC). The L segment encodes the viral polymerase (L protein) and RING finger Z matrix protein. The precursor GPC forms the membrane-bound GP1 and GP2 subunits via post-translational cleavage by the protease SKI-1/S1P [13]. GP1 serves a putative role in receptor binding, while the structure of GP2 is consistent with viral transmembrane fusion proteins [14]. NP binds and protects the viral RNA in the virion [15-17]. The Z matrix protein associates with GP2 and NP during viral biogenesis, but alone is sufficient to mediate formation and release of viral particles from infected/transfected cells [18-21].

## Results

### Differential detection of LASV antigens in infected human patient sera

**Figure 1:**
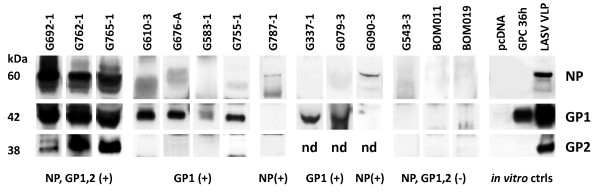
**Western blot analysis of LASV antigen positive and negative patient sera and controls for GP1, GP2, and NP proteins**. Twenty μL of a 1:4 dilution of precipitated suspected LASV patient sera were resolved per lane of a reducing SDS-PAGE gel, blotted and probed with α-GP1, α-GP2, or α-NP antibodies. In three samples NP, GP1, GP2 were detected, indicating presence of LASV virions (G692-1, G762-1, G765-1; NP, GP1,2 (+)). In G610-3, G676-A, G583-1, and G755-1 only GP1 was detected (GP1 (+)). Only low levels of NP were detected in G787-1 (NP (+)). Whereas in G337-1 and G079-3 only GP1 was detectable (GP1 (+)), GP2 levels were not determined (nd). Similarly, in G090-3 only low levels of NP were detected, but GP2 levels were not determined (nd). A representative suspect LASV patient serum sample that did not reveal detectable levels of all three viral antigens (G543-3) is shown alongside normal uninfected controls (BOM011, BOM019) (NP, GP1,2 (-)). *In vitro *controls derived from transfection of HEK-293T/17 cells with pcDNA vector (pcDNA) or a LASV GPC construct harvested at 36 hours (GPC 36 h) are shown. Soluble GP1 can be precipitated from the supernatants of cells expressing GPC (GPC 36 h). LASV VLP containing NP, GP1, and GP2 are shown for protein size comparison (L VLP) (*in vitro *ctrls). Molecular weights for each LASV protein are shown to the left of blots, and identified on the right.

A total of 19 and 27 patient sera were analyzed in the first and second studies, respectively. A panel of serum samples from approximately 100 volunteer blood donors from the Northwestern district of Bombali, Sierra Leone, designated BOM series, were analyzed for LASV-specific antigen and IgG and IgM antibodies. These samples were collected from individuals whose reported medical histories did not contain indication of previous infection with Lassa virus. A significant percentage of BOM samples tested positive for IgM and IgG antibodies against LASV proteins in a recombinant ELISA, but all 100 were negative for virus antigen in a capture assay using an NP-specific detection platform (data not shown). Additionally, a panel of archived and freshly collected human sera from patients that had been admitted to the KGH from January 2008 - April 2010 as suspected Lassa fever cases were chosen for these studies. All archived samples containing sufficient quantities of serum (~ 20 - 25 μL) with a diagnosis of "antigen positive" (Ag+) determined by a traditional Ag-capture assay (Trad Ag) developed by the United States Army Medical Research Institute of Infectious Diseases (USAMRIID), a recombinant Ag-capture assay for LASV NP developed by the Hemorrhagic Fever Virus Diagnostics Consortium, or from patients presenting with classical clinical symptoms of Lassa fever were used in this study. In addition, a subpanel of samples that were Ag-negative but IgM and/or IgG positive, either by traditional LASV antibody platform (USAMRIID) [Trad IgG, Trad IgM] or recombinant protein-based (Hemorrhagic Fever Virus Diagnostics Consortium) [r IgG, r IgM] ELISA, were analyzed as controls. A profile of LASV antigens detected in serum samples analyzed in these studies is displayed in figure 1. All three antigens, NP, GP1, and GP2, were detected at high levels in only three patient sera (figure 1, G692-1, G762-1, G765-1). The former two patients succumbed while the outcome of G765-1 is unknown. In six independent patient sera, GP1 antigen was the only antigen detected (figure 1, G610-3, G676-A, G583-1, G755-1, G337-1, G079-3), albeit at relatively low levels compared to the triple Ag-positive samples. Samples G337-1 and G079-3 were not analyzed for presence of the GP2 protein due to scarcity of archived sera (figure 1). In two samples only, NP antigen could be detected at low levels (figure 1, G787-1, G090-3); however, GP2 analysis was not performed for the latter. In 34 patient and control sera, none of the three LASV proteins were detected, as outlined in the three examples in figure 1 (figure 1, G543-3, BOM011, BOM019). Identification of LASV proteins NP, GP1, and GP2 in patient sera was aided by comparing corresponding bands on VLP (figure 1, LASV VLP). The 42 kDa sGP1 component generated from expression of GPC in HEK-293T/17 cells was precipitated from the supernatant with PEG and salt and readily detected (figure 1, GPC 36 h). Viral proteins could not be detected in precipitated supernatants from cells transfected with plasmid vector (figure 1, pcDNA).

**Figure 2:**
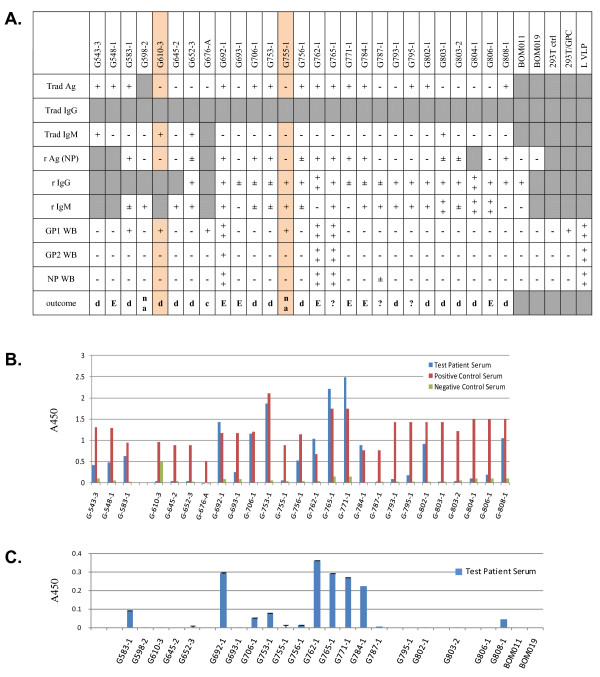
**Antigen, IgG, IgM, and outcome profiles in suspected LASV patient sera from the first study (December 2009): G079-3 through G617-1**. A. Trad Ag, IgG, IgM (USAMRIID), and r Ag, IgG, IgM (Hemorrhagic Fever Virus Diagnostics Consortium) were ELISA platforms, whereas GP1, GP2, NP WB were western blots. The USAMRIID assays were performed before the recombinant counterparts, sometimes months in advance. Western blot analyses were performed on samples shipped to the U.S. following irradiation. A very strong positive signal is indicated by ++ and a positive by +. Marginal positive detection is indicated by +/- and negatives by -. Gray boxes indicate that relevant data was not collected for the corresponding sample. Patient outcomes recorded in databases are: d, discharged; D, dead; na, not admitted; c, household contact of patient G676; ?, patient samples from Liberia, with unknown outcome. Non applicable (N/a) outcome is indicated for *in vitro *controls. B. Traditional antigen (Trad Ag) data showing the A450 O.D. for all samples (blue) alongside positive (dark red) and negative (green) controls. Data are plotted as mean ± SD, n = 2. Each sample in this series was analyzed on different days, concurrent with testing of suspect patients admitted to KGH. Collection dates for this series ranged from January 2008 to September 2009.

**Figure 3:**
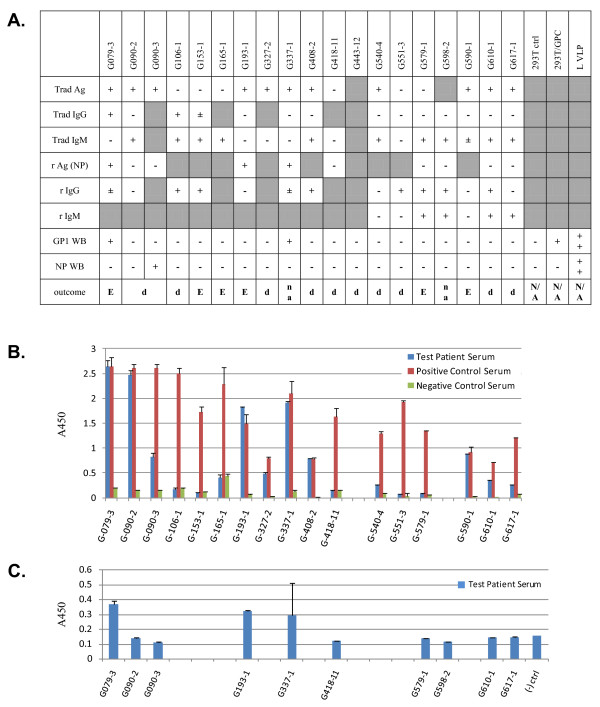
**Antigen, IgG, IgM, and outcome profiles in suspected LASV patient sera from the second study (March and April 2010): G543-3 through G806-1**. A. The legend and designations outlined in figure 2A above apply to figure 3. Sample G598-2 was duplicated in this series to verify previous results. B. Traditional antigen (Trad Ag) data as outlined for figure 2B above. Data are plotted as mean ± SD, n = 2. Each sample in this series was analyzed on different days, concurrent with testing of suspect patients admitted to KGH. Collection dates for this series ranged from June 2009 to March 2010.

In figures 2 and 3 western blot results were compared to available data generated with recombinant and traditional LASV antigen capture, IgM, and IgG assays. Figure 2A displays western blot data for GP1, GP2, and NP, whereas figure 3A lacks GP2 analysis. Antigen capture data using the traditional assay platform are displayed in figures 2B and 3B, and contain averaged A450 absorbance values for test patient, positive, and negative control sera. For each set of samples, the data were not normalized due to the lack of internal assay calibrators and analysis of patient sera on different dates concurrent with collection and analysis at the KGH. In these studies, presence of sGP1 as the sole detectable arenaviral antigen in serum samples was established only when both GP2 and NP could not be equally detected. Exclusionary parameters for sole detection of sGP1 were positive results in a traditional capture assay employing GP1, GP2, and NP-specific mAb cocktails, in a recombinant NP antigen capture platform, and detection of other viral components in western blots, as outlined in methods. The only two samples that met these criteria in the current studies were G610-3 and G755-1 (figure 1, 2A, 2B, 2C).

## Discussion

In previous studies we described and characterized the phenomenon of LASV GP1 ectodomain shedding *in vitro *[1,2]. In order to investigate whether this phenomenon was specific *in vivo*, we analyzed serum samples from patients admitted to the KGH in Sierra Leone from 2008 - 2010 for the differential detection of LASV proteins. Our approach involved the detection of GP1, GP2, and NP in the same sample, with sensitive monoclonal and polyclonal antibodies. The detection of NP alone could be indicative of release of the protein from virally infected dead cells, and could conceivably remain in the bloodstream after viral clearance. This phenomenon has been observed *in vitro *[16,22]. Thus, acute viremia was characterized as the concomitant detection of all three viral proteins, GP1, GP2, and NP. The detection of GP2 implies its presence in the context of an enveloped virion, due to its known membrane-spanning properties via the transmembrane domain (amino acids 427 - 451 in LASV Josiah). Similarly, the presence of GP1 in the same samples would imply viremia because it associates with GP2 as a component of the non-covalently linked tripartite GPC complex. The nucleoprotein component of the virion should also be detected in all samples containing GP1 and GP2, thus confirming the presence of intact, enveloped, circulating Lassa virions. The detection of only GP1 in any given sample was interpreted as the absence of whole virions and the presence of the soluble form of the protein, as previously observed *in vitro*. The date and time of collection of any given blood sample represents a snapshot in the stage of a potential LASV infection. Within the context of an early acute viral infection, it is unlikely that a patient would present with symptoms immediately following exposure to the virus. Following viral infection of host cells and early replication events, the detection of sGP1 without accompanying progeny virions might be possible. This event may represent a very narrow window in the virus life cycle before the emergence of mature virions from infected cells *in vivo*. Thus, the ratio of samples where sGP1 alone was conclusively detected in the context of these studies was small (2/46). Following this very early step in viral biogenesis where host cells are secreting only sGP1, progeny virions will emerge from the surface of infected cells and will disseminate throughout body tissues and fluids. At this stage, differentiation of sGP1 from virion-associated GP1 is no longer possible. In the cases where any viral antigens, IgM and IgG were detected (G692-1, G762-1, G765-1), the possibility exists that a re-infection with clinical symptoms and development of febrile disease occurred. Two out of these three patients succumbed to Lassa fever; the outcome of patient G765-1 is not known. Each sample analyzed in these studies for LASV antigens was exhaustively subjected to western blot analysis with different viral protein-specific antibody reagents, at different serum dilutions, with extended exposures to sensitive films, and using an extensive panel of positive and negative controls to ensure that data were not the result of artefacts or background effects. Therefore, each sample was analyzed for presence of each antigen at least three times, either in a primary detection or in probing and reprobing formats. Although probing and reprobing allowed for the detection of antigens using a single blot, and thus preserving precious sample volumes, the membrane stripping process removes protein from the matrix, thus reducing the sensitivity of subsequent assays. The small volumes of available serum for analysis made further characterization of each sample unfeasible. Future studies will be aimed at characterizing the nature of shed sGP1 *in vivo*, namely proteolysis sensitivity and deglycosylation analyses, for which relevant *in vitro *data is available.

In these studies three sets of relevant data were used to interpret the antigenic status of the serum samples: 1. Traditional antigen (Trad Ag) ELISA; 2. Recombinant antigen capture, NP-specific; 3. Western blot analysis of GP1, 2 and NP. Direct correlations cannot be made between the three assay platforms based on several factors. The USAMRIID antigen capture assay employs two GP1-, two GP2-, and one NP-specific mAbs [24]. Thus, individual identification of each antigen cannot be established. Furthermore, this assay relies on a two-step signal amplification and detection, each with polyclonal antibody reagents, a rabbit secondary and a goat tertiary. The sensitivity of this assay, therefore, may be superior to the recombinant NP mAb-based antigen capture format, which employs a single detection step with a goat polyclonal raised against recombinantly expressed NP protein. Both antigen capture formats were performed with 1:10 dilutions of serum in assay buffer, in a total of 100 μL. Then, western blots were performed with precipitated protein from 5 μL of serum. Considering that each analytical format detects antigens in different conformations, direct data comparison is not feasible. Despite specificity and sensitivity issues at play, in 21/30 samples for which data is available from all three assay formats, the results from the Trad Ag and r Ag platforms agreed. In six samples, the Trad Ag assay detected LASV antigen and the r Ag platform did not. The higher sensitivity of the former may be due to the combination of 3 antigen-specific mAbs that detected LASV glycoproteins in addition to NP in the Trad Ag assay. Another possibility to consider is the quality of stored samples in less than ideal conditions over extended periods of time. The lack of continuous electrical power at the KGH over the years has resulted in temperature fluctuations in refrigerators and freezers, which may interfere with sample quality and stability of viral antigens. The time that elapsed between collection and analysis of samples used in these studies varied between 3 months and 2 years. Despite these technical issues, many samples that originally tested positive for antigen, IgM, and IgG, retain the specific binding pattern irrespective of the time elapsed since collection. A complete list of samples, dates of collection, antigen status, and known storage temperature are shown in Table 1. The two samples in which only sGP1 could be detected in these studies (G610-3 and G755-1) were collected in September 2009 and January 2010, and thus analyzed within 3 months of collection, in December 2009 and April 2010, respectively.

**Table 1:** Collection and sample processing dates for G-series samples

Sample ID	Date of collection	Date processed	Comments	rAg status	Sample storage temperature
G079-3	16-Jan-08	December 2009	Expired	NP, GP1	+10°C to -5°C, fluctuating w/power availability at KGH LFL
			
G090-2	01-Feb-08		Discharged		
				
G090-3	02-Feb-08			NP (WB)	
			
G106-1	19-Feb-08		Discharged		
			
G153-1	07-May-08		Expired		
			
G165-1	15-May-08		Expired		
			
G193-1	16-Jun-08		Expired	NP (ELISA)	
			
G327-2	17-Dec-08		Discharged	NP (ELISA), GP1	
			
G337-1	12-Jan-09		Not admitted		
			
G408-2	12-Feb-09		Discharged		
			
G418-11	22-Jul-09		Follow-up		
			
G443-12	Apr-09		Follow-up		
			
G540-4	May-09		Discharged		
			
G543-3	24-Jun-09		Discharged		
			
G548-1	21-Jun-09		Expired		
			
G551-3	28-Jun-09		Discharged		
			
G579-1	13-Jul-09		Expired		
			
G583-1	16-Jul-09		Discharged	NP (ELISA), GP1	
			
G590-1	24-Jul-09		Expired		
			
G598-2	19-Aug-09		Not admitted		
			
G610-1	27-Aug-09		Discharged		
				
G610-3 *	03-Sep-09			GP1	
			
G617-1	15-Sep-09		Discharged		
			
G645-2	24-Oct-09		Discharged		
			
G652-3	02-Nov-09		Discharged	NP (ELISA, ±)	
			
G676-A	Dec-09		Contact of G676	GP1	

G692-1	31-Dec-09	April 2010	Expired	NP (ELISA, WB), GP1, GP2	-20°C, consistent temperature, power supplied by solar panel array 24 hours/day
			
G693-1	31-Dec-09		Expired		
			
G706-1	07-Jan-10		Discharged	NP (ELISA)	
			
G753-1	27-Jan-10		Discharged	NP (ELISA)	
			
G755-1 *	27-Jan-10		Not admitted	GP1	
			
G756-1	27-Jan-10		Discharged	NP (ELISA,±)	
			
G762-1	29-Jan-10		Expired	NP (ELISA, WB), GP1, GP2	
			
G765-1	06-Feb-10		Unknown outcome	NP (ELISA, WB), GP1, GP2	
			
G771-1	08-Feb-10		Expired	NP (ELISA)	
			
G784-1	17-Feb-10		Expired	NP (ELISA)	
			
G787-1	18-Feb-10		Unknown outcome	NP (WB, ±)	
			
G793-1	24-Feb-10		Discharged		
			
G795-1	25-Feb-10		Unknown outcome		
			
G802-1	25-Feb-10		Discharged		
			
G803-1	25-Feb-10		Discharged	NP (ELISA)	
			
G803-2	27-Feb-10		Discharged	NP (ELISA)	
			
G804-1	26-Feb-10		Discharged		
			
G806-1	25-Feb-10		Expired		
			
G808-1	27-Feb-10		Discharged	NP (ELISA)	
			
BOM011	Apr-10		Healthy volunteer		
			
BOM019	Apr-10		Healthy volunteer		

Nine samples tested positive for sGP1 by western blot (G079-3, G337-1, G583-1, G610-3, G676-A, G692-1, G755-1, G762-1, G765-1). In three additional samples, the r Ag assay marginally detected NP in serum samples (G652-3, G803-1, G803-2). However, these three samples did not test positive for sGP1 or NP by western blot. Our data suggests that a NP-based Ag capture assay currently in use has a sensitivity threshold of 1.5 ng rNP/mL (unpublished data). Western blots have not thus far successfully detected NP Ag in the low ng/mL level. Three other samples tested positive in the Trad Ag and r Ag ELISA assays, along with detection of sGP1 by western blot analysis, but did not test positive for NP by western blot, and were, therefore, not considered examples of glycoprotein shedding (G079-3, G337-1, G583-1). In this group of samples only G583-1was analyzed for presence of GP1, GP2, and NP, whereas the remaining two were not tested for GP2. Despite the absence of GP2 by western blot, the presence of NP by antigen capture ELISA and the fact that it was positive by the traditional antigen capture ELISA made it impossible to distinguish between the phenomenon of sGP1 shedding and the presence of GP1 in enveloped virion format. Thus, we have considered samples G610-3 and G775-1 as the only examples of clear LASV GP1 shedding in these studies, though the phenomena of GP1 shedding may occur throughout viremia. In both samples, LASV antigen was not detected by Trad Ag and r Ag ELISA, and western blot did not reveal presence of GP2 and NP. However, GP1 was clearly present (figure 1, G610-3, G755-1). For sample G676-A, collected from a household contact of patient G676 who succumbed to Lassa fever, Trad Ag and r Ag ELISA data were not available; therefore, this sample was also not considered a clear example of GP1 shedding.

Based on the proposed narrow window of sGP1 detection *in vivo *likely to occur in the first few days after primary infection with LASV, it is not surprising that only 2/46 samples analyzed in these studies would not show any other viral proteins indicating an early LASV infection. The known incubation period for infection with LASV is approximately 6 - 7 days [24,25]. However, admission to local hospitals is usually delayed following the onset of febrile disease compounding the low detection rate of shed GP1. Thus, in many cases, patients admitted to the KGH present with acute viremia, or undetectable antigen but rising IgM titers, indicative of advanced LASV infection.

**Figure 4:**
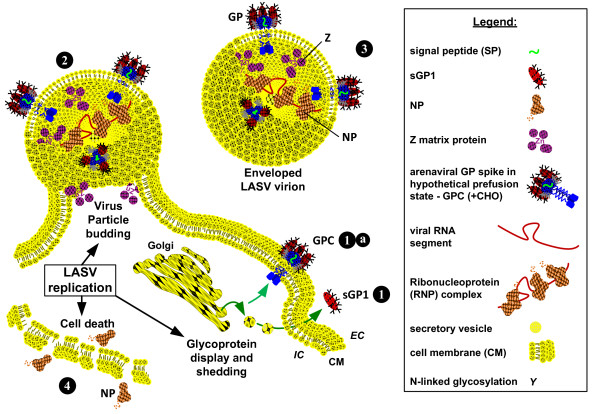
**Graphic model of glycoprotein display and sGP1 shedding, relevant protein components in LASV virions, and release of viral components from dead cells**. (1) LASV glycoprotein mediates virion binding to alpha dystroglycan (αDG) on permissive cells (1a). Following receptor binding the virion fuses with the host cell membrane, resulting in viral entry. Viral replication takes place in the cytoplasm of the infected cell. (2) During replication and before mature virions are assembled GPC is proteolytically processed and a portion of the resulting GP1 is transported to the cell surface and shed as sGP1 (2a). Concurrently, GPC is expressed on the cell surface, mediated by GP2 anchoring in the bilipid membrane, non-covalently associated GP1, and the hydrophobic signal peptide (2b). (3) Budding virions emerge from the cell surface mediated by Z matrix protein as enveloped particles containing membrane lipid and proteins, viral GPC and nucleoprotein. (4) Virions are released from infected cells containing nucleoprotein and associated viral RNA segments, along with non-displayed polymerase and cellular ribosomes. Budded virions will enter the circulation and can be detected in whole antigen capture assays or by western blot identification of individual proteins, as performed in these studies. Presence of intact virions should permit the identification of GP1, GP2, and nucleoprotein. (5) Nucleoprotein and glycoproteins can shed from dead or dying cells, with compromised cell membrane integrity. This phenomenon is observed *in vitro*, and may occur *in vivo*, hence the detection of only NP in some serum samples. A graphic representation of each relevant component in the outlined pathway is shown in the legend box. EC: extracellular; IC: intracellular; CM: cell membrane.

Strecker et al., 2003 [19] reported the stoichiometric ratio of NP:GP1:GP2 in Lassa virions as 160:60:60. In the context of a LASV virion, detection of one protein should result in the detection of the other two. Thus, detection of GP1 without concomitant detection of either GP2 or NP can be interpreted as a secreted isoform of GP1 prior to but not necessarily limited to the emergence of enveloped viral particles from infected cells, that likely corresponds to the sGP1 component identified *in vitro *(figure 1, GPC 36 h) [1,2]. Although the possibility exists that sole detection of sGP1 in serum samples is the result of degraded virions or viral protein released from infected cells, recent studies with LASV VLP suggest differently. The structure and function of arenaviral glycoproteins have resulted in significant resistance to proteolytic digestion with highly active proteases, namely trypsin and Proteinase K [26]. We and others have observed that glycosylated LASV GP1 and GP2 are highly resistance to the effects of proteolytic digestion, whereas NP and Z are not. Thus, degraded virions that afford detection of GP1 should also result in equivalent detection of GP2, despite the loss of NP (and Z). Moreover, lysis of infected cells *in vivo *would parallel the effects of *in vitro *LASV infection, but selective release of the GP1 protein is highly unlikely. If GP1 generated during viral biogenesis is released from lysed cells, concomitant detection of GP2 and NP should be observed. Generation of LASV VLP in mammalian cells via transfection with GPC, NP, and Z gene constructs resulted in pseudoparticles containing all three proteins closely associated in enveloped structures. Additionally, sGP1 and a minor soluble NP fraction, but not Z or GP2, could be detected in the least dense sucrose fraction in gradient centrifugation assays, indicating their lack of association with enveloped pseudoparticles [26]. Release of NP from dead cells was observed in the studies by Branco et al. [26] and by and others [22]. Conversely, soluble GP1, but not GP2, could be readily detected in the supernatant as early as 12 hours after transfection with GPC constructs *in vitro*. Release of NP from transfected cells was not observed in the context of single gene transfections [26]. Together, these results strongly suggest that a soluble GP1 component can be secreted from cells expressing the LASV glycoprotein complex prior to release of mature virions *in vivo*. A graphic model outlining a proposed temporal progression of virus infectivity of permissive cells, glycoprotein display and sGP1 shedding, relevant protein components in LASV virions, and release of viral components from dead cells is shown in figure 4.

## Conclusions

Through this work, we have identified a soluble form of LASV GP1 in the serum of infected patients. Although the exact stage of viral infection in all patients could not be determined through these studies, the lack of detection of the virion associated NP and GP2 proteins, with clear identification of GP1 in the serum of acutely infected individuals, points to an early event in viral replication when only sGP1 can be detected. Although a role for sGP1 in arenaviral infections *in vivo *has not been established, future work will attempt to characterize functional roles for this protein. It is likely that LASV sGP1 performs immunomodulatory and decoy functions similar to those proposed for EBOV, a filovirus; respiratory syncytial virus (RSV), a paramyxovirus; human immunodeficiency virus (HIV), a retrovirus; and rabies virus (RABV), a rhabdovirus. In EBOV and RSV infections it has been proposed that ectodomain shedding of the viral envelope glycoprotein serves as an immune decoy, with significant enhancement of pathogenicity *in vivo *[3,27].

Definition of the role(s) of LASV sGP1 *in vivo *could lead to new correlates of the disease, opportunities toward development of diagnostics targeting early events in acute infection and viral biogenesis, and the ability to counter potential viral immunomodulatory pathways that confer poor outcomes.

## Methods

### Human serum collection

Febrile patients suspected of having LF were admitted to the LF Ward at the Kenema Government Hospital (KGH) in Kenema, Sierra Leone. Informed consent was obtained and each patient was assigned a unique identification number (G-XXXX). Approximately 5-10 ml of blood was collected from each patient. Blood was collected into 5 ml serum collection tubes using butterfly safety needles. The same procedures and assurances were employed in the collection of blood from normal human volunteers. Normal human sera used in these studies were collected in the Bombali district of Northern Province, Sierra Leone, and designated BOM.

### Precipitation of total protein from human serum samples

Serum samples collected from patients admitted to the KGH Lassa fever ward (G-series), household contacts of hospitalized subjects (G-series-A), and individuals not known to have had Lassa fever (BOM) were aliquoted and stored in cryovials at the KGH Lassa fever laboratory. Samples used in these studies were visually inspected for presence of follicular precipitate, coagulate, significant discoloration, contamination, and haemolysis. Only clear serum samples were used. Twenty μL of each serum sample were diluted 5-fold with sterile D-PBS, pH 7.4 and combined with 20% polyethylene glycol-6000 (PEG-6000) and 2 M NaCl stock solutions to final concentrations of 5% and 0.2 M, respectively. Samples were incubated at 4°C overnight, followed by centrifugation at 21,000 ×*g*, for 75 minutes at 4°C. Supernatants were carefully aspirated and discarded. Pellets were resuspended in SDS-PAGE buffer with 50% glycerol, heated without reducing agent, and stored frozen until shipment. Samples were shipped to the U.S. in IATA-approved containers and were irradiated with 2500 KRad upon arrival, using a Cs source. Recombinant LASV VLP expressing Z+NP+GPC were used as controls for identification of viral proteins in SDS-PAGE, along with soluble GP1 (sGP1) from HEK-293T/17 cells transfected with a wild type GPC gene. The generation of LASV VLP and sGP1 have been described elsewhere [1,2,26].

### Western blot analysis

Four-fold dilutions of protein sample from a 20 μL serum aliquot were prepared in SDS-PAGE sample buffer, reduced with DTT, heated to 75°C for 10 minutes, and resolved on 10% NuPAGE Novex Bis-Tris gels, according to the manufacturer's specifications (Novex, San Diego, CA). Proteins were transferred to 0.45-μm nitrocellulose membranes, blocked, and probed in 1X PBS, pH 7.4, 5% non-fat dry milk, 1% heat inactivated fetal bovine serum, 0.05% Tween-20, and 0.1% thymerosal. Detection of LASV GP1, GP2, and NP in precipitated protein from human serum samples was performed by Western blot analysis using anti-LASV mAbs L52-74-7A (GP1), L52-216-7 and L52-272-7 (GP2), and goat PAb to *E. coli *generated nucleoprotein, respectively. Secondary antibodies were horseradish peroxidase (HRP)-conjugated goat anti-mouse IgG (H+L) or rabbit anti goat IgG (H+L). Membranes were then incubated in LumiGlo chemiluminescent substrate (KPL) and exposed to HyBlot CL Film (Denville Scientific, Inc). Blots used in reprobing experiments were briefly rinsed in PBS-T (1X PBS, pH 7.4, 0.1% Tween 20) after exposure to x-ray film, followed by incubation in stripping buffer (62.5 mM Tris, pH 6.8, 2% SDS, 100 mM β-ME) for one hour at 65°C. Blots were then washed extensively in PBS-T, re-blocked, and reprobed as outlined above. Blots were reprobed a maximum of three times.

## Competing interests

Luis M Branco, Frederick J Geske, and Robert F Garry are listed inventors, in addition to others, in a PCT application entitled "Soluble and Membrane-Anchored Forms of Lassa Virus Subunit Proteins", filed in April 2008. Additionally, Luis M Branco and Robert F Garry are listed inventors in a provisional application for United States letters patent entitled "Lassa virus particles and methods for production thereof", filed in September 2009. This work was performed as partial fulfilment of Ph.D. dissertation requirements for Luis M Branco and Jessica N Grove.

## Authors' contributions

LMB and JNG contributed to the experimental design, performed sample analysis, interpreted data, and drafted the manuscript. LMM coordinated blood sample collection from LASV convalescent individuals according to established IRB protocols, managed the KGH outreach team, and helped process samples. AG, MF, and MM coordinated blood sample collection from convalescent individuals and from suspected Lassa fever patients at the KGH, performed and managed the analysis of sera by traditional antigen, IgM, and IgG assays. RJS kindly provided monoclonal antibodies to relevant LASV proteins, and critically reviewed the manuscript. DB provided critical review of the manuscript. RFG contributed to the experimental design and provided critical review of the manuscript.
